# Correction to: ‘Sex differences in human mate preferences vary across sex ratios’ (2023) by Walter *et al.*

**DOI:** 10.1098/rspb.2023.1513

**Published:** 2023-07-26

**Authors:** Kathryn V. Walter, Daniel Conroy-Beam, David M. Buss, Kelly Asao, Agnieszka Sorokowska, Piotr Sorokowski, Toivo Aavik, Grace Akello, Mohammad Madallh Alhabahba, Charlotte Alm, Naumana Amjad, Afifa Anjum, Chiemezie S. Atama, Derya Atamtürk Duyar, Richard Ayebare, Carlota Batres, Mons Bendixen, Aicha Bensafia, Boris Bizumic, Mahmoud Boussena, Marina Butovskaya, Seda Can, Katarzyna Cantarero, Antonin Carrier, Hakan Cetinkaya, Ilona Croy, Rosa María Cueto, Marcin Czub, Daria Dronova, Seda Dural, Izzet Duyar, Berna Ertugrul, Agustín Espinosa, Ignacio Estevan, Carla Sofia Esteves, Luxi Fang, Tomasz Frackowiak, Jorge Contreras Garduño, Karina Ugalde González, Farida Guemaz, Petra Gyuris, Mária Halamová, Iskra Herak, Marina Horvat, Ivana Hromatko, Chin-Ming Hui, Jas Laile Jaafar, Feng Jiang, Konstantinos Kafetsios, Tina Kavčič, Leif Edward Ottesen Kennair, Nicolas Kervyn, Truong Thi Khanh Ha, Imran Ahmed Khilji, Nils C. Köbis, Hoang Moc Lan, András Láng, Georgina R. Lennard, Ernesto León, Torun Lindholm, Trinh Thi Linh, Giulia Lopez, Nguyen Van Luot, Alvaro Mailhos, Zoi Manesi, Rocio Martinez, Sarah L. McKerchar, Norbert Meskó, Girishwar Misra, Conal Monaghan, Emanuel C. Mora, Alba Moya-Garófano, Bojan Musil, Jean Carlos Natividade, Agnieszka Niemczyk, George Nizharadze, Elisabeth Oberzaucher, Anna Oleszkiewicz, Mohd Sofian Omar-Fauzee, Ike E. Onyishi, Baris Özener, Ariela Francesca Pagani, Vilmante Pakalniskiene, Miriam Parise, Farid Pazhoohi, Annette Pisanski, Katarzyna Pisanski, Edna Ponciano, Camelia Popa, Pavol Prokop, Muhammad Rizwan, Mario Sainz, Svjetlana Salkičević, Ruta Sargautyte, Ivan Sarmány-Schuller, Susanne Schmehl, Shivantika Sharad, Razi Sultan Siddiqui, Franco Simonetti, Stanislava Yordanova Stoyanova, Meri Tadinac, Marco Antonio Correa Varella, Christin-Melanie Vauclair, Luis Diego Vega, Dwi Ajeng Widarini, Gyesook Yoo, Marta Marta Zaťková, Maja Zupančič

*Proc. R. Soc. B* **288**, 20211115 (Published online 21 July 2021). (https://doi.org/10.1098/rspb.2021.1115)

The first affiliation listed under author Katarzyna Pisanski is incorrect: Department of Psychology, Westminster College, Salt Lake City, UT 84105, USA.

The correct first affiliation is:

Institute of Psychology, University of Wroclaw, Wroclaw 50137, Poland

Note that second and third affiliations are correct.

The original has now been corrected.

